# Psychological distress among people living with chronic medical illness and the general population, Northwest Ethiopia: A comparative cross-sectional study

**DOI:** 10.1371/journal.pone.0278235

**Published:** 2022-12-01

**Authors:** Minale Tareke, Agegnehu Berie Bayeh, Minyichil Birhanu, Amsalu Belete

**Affiliations:** 1 Department of Psychiatry, College of Medicine and Health Sciences, Bahir Dar University, Bahir Dar, Ethiopia; 2 Department of Surgery, College of Medicine and Health Sciences, Bahir Dar University, Bahir Dar, Ethiopia; 3 Department of Paediatrics and Child Health Nursing, College of Medicine and Health Sciences, Bahir Dar University, Bahir Dar, Ethiopia; 4 Department of Psychiatry, College of Medicine and Health Sciences, Debre Tabor University, Debre Tabor, Ethiopia; Al-Jouf University College of Pharmacy, SAUDI ARABIA

## Abstract

**Background:**

Psychological distress is often linked to and negatively affects the outcomes of chronic medical conditions; however, data on psychological distress and its predictors among individuals with chronic medical illnesses are scarce in developing countries like Ethiopia. The main objective of this study was to assess the magnitude and predictors of psychological distress among people living with chronic medical illness and the general population.

**Methods:**

A comparative cross-sectional study was conducted in Mecha demographic research center, Northwest Ethiopia. The participants were selected from the general population and outpatient departments. Kessler-10 (K-10) was used to assess psychological distress.

**Result:**

The magnitude of psychological distress among people living with chronic medical illness and those from the general population was 62.0% and 35.1%, respectively. The odds of psychological distress in people living with chronic medical illness was three times more than the one in the general population. Divorced marital status, family history of chronic medical illness, and low social support were statistically significantly associated with psychological distress in both groups.

**Conclusion:**

The magnitude of psychological distress was significantly higher among people living with chronic medical illness. Routine screening of such cases for psychological distress during their visits for their medical illness helps take appropriate therapeutic interventions.

## Introduction

Mental disorders, which include psychological distress remained at the top of the global burden of disease. This has been highly worsened by the recent COVID-19 pandemic [1, 2]. Psychological distress is a state of emotional suffering characterized by symptoms of depression and anxiety which often co-exist with somatic complaints. It is a highly prevalent and disabling condition affecting about 10% of the world’s population once in their lifetime [3]. The 2010 Global Burden of Disease Study showed that major depression was the second leading cause of disability and a major contributor to the burden of suicide and ischemic heart disease [4, 5].

Three-quarters of individuals affected by psychological distress are found in low-income countries [6]. It is considered a silent epidemic throughout Africa due to substantial financial and systemic challenges [7]. Many factors are known to be associated with mental health problems [8]. Psychological distress is influenced by demographic factors, such as age, gender, lowest socioeconomic status, poorer physical health, low educational status, homelessness, and unemployment [4, 6, 9].

An epidemiological study done on mental disorders in Kenya found a 10.8% prevalence rate with no gender difference. Significant risk factors were age and the presence of physical illness [10]. A community-based cross-sectional study conducted in Northeast Ethiopia showed that the prevalence of common mental disorders was 32.4%. Being female, no formal education and low level of education, having a small family size, family history of mental illness, living with chronic illness, active smoking, and experiencing one or more stressful life events were significantly associated with common mental disorders. On the other hand, a high level of emotional support reduces the likelihood of occurrence of psychological distress by half [11].

Psychological distress is often linked to and worsens the outcomes for chronic diseases such as cancer, cardiovascular diseases, diabetes, tuberculosis, and HIV/AIDS due to its effect to have unhealthy and risky behavior, non-adherence to prescribed medical regimens, and diminished immune functioning [5]. Untreated psychological distress resulted in poor control of sugar level in diabetes mellitus; worse treatment outcomes, poorer quality of life and greater disability in tuberculosis; uncontrolled seizure in epilepsy; and high blood pressure in hypertension [12–15]. However, the magnitude and predictors of psychological distress among people living with chronic medical illness in low-income countries like Ethiopia remain under-researched. Hence, this study aimed at determining the magnitude and identifying predictors of psychological distress among people living with chronic medical illness in comparison with the general population in Northwest Ethiopia. The research hypothesis was that psychological distress was more prevalent among people living with chronic medical illness than in the general population. The findings of the study help increase the health care providers’ awareness of the magnitude of psychological distress and its predictors among both groups of the population. This, in turn, leads to early diagnosis and treatment of mental distress and improves treatment outcomes and quality of life.

## Materials and methods

### Study design, setting, and period

A comparative cross-sectional study was conducted in Mecha demographic surveillance and field research center catchment areas in Mecha district, Northwest Ethiopia from March to April 2018. A facility-based sample of individuals who were diagnosed with chronic medical diseases (hypertension, tuberculosis, diabetes mellitus, HIV/AIDS, epilepsy, and asthma) was compared to a community-based representative sample of healthy individuals.

### Target population

The target population included all individuals living in the Mecha district for at least six months, the minimum time duration one needs to qualify to be a resident in the district under local regulations.

### Inclusion and exclusion criteria

#### People living with chronic medical illness

All adults aged 18 years and above who have treatment follow-up for chronic medical illnesses at health facilities in the study district for at least 6 months were included in the interview.

#### Participants from the general population

All people aged 18 years and above who have been living in the area for at least six months were invited for the interview. Those who reported known chronic medical illnesses were excluded to control a potential confounding effect.

### Sample size and sampling procedures

The sample size was calculated using Epi info StatCalc for a comparative cross-sectional study with a confidence interval of 95%, statistical power of 80%, level of significance of 5%, psychological distress among people attending outpatient departments for chronic diseases, and among community dwellers of 42.4% [13] and 32.4% [11], respectively with equal ratio. The sample size calculated was 387 for each of the two groups. A design effect of two was used to calculate the actual final sample size. A total of 1548 respondents were recruited for both groups.

The government health facilities in the study area were selected with a lottery method. The list of all catchment kebeles (districts) for the selected health facilities were obtained. The multistage sampling went on by taking a sample of Kebeles, villages, and then households using a lottery method. Proportional allocation of the calculated sample size to the selected villages and health facilities was done based on the proportion of people living with chronic medical illness in the health facilities. Finally, participants from the selected households and health facilities were recruited by a systematic random sampling.

### Data collection tools, procedures, and measurements

Six data collectors and two supervisors were selected and given a two-day training. With daily supervision, the data was collected via face-to-face interview using a pre-tested structured questionnaire consisting of socio-demographic and psychosocial variables.

Socio-demographic variables included sex, age (in years), marital status, educational status, occupation, and monthly income (in Ethiopian birr, quartiles).

The psycho-social variables were family history of medical illness (yes, no) and mental illness (yes, no), family size, living condition (lives with family, relatives, alone, homeless), types of drugs (one, two, or more), duration of medication (0–5 years, > 5 years), stressful life events, and social support.

Psychological distress was assessed using K-10, a 10-item scale validated in Ethiopia and found in the Amharic version. It has a 5-point Likert scale from zero to four: 0 (none of the time), 1 (a little of the time), 2 (some of the time), 3 (most of the time), and 4 (all of the time). The total score for an individual ranges from zero to forty [16]. It measures the psychological distress experienced by people in the last 30 days before the study [17]. It has very good psychometric properties, 84.2% sensitivity and 77.8% specificity at a cut-off threshold of a minimum of 7 out of 40 [16, 18]. This cut-off point was used in this study to dichotomize psychological distress. A Cronbach’s alpha (0.94) demonstrated a high level of internal consistency for the K-10 items in this study.

Stressful life events were assessed using the List of Threatening Events (LTE), a 12-item self-report questionnaire. Participants indicated if these life events occurred in the six months before the data collection giving a total score of zero to twelve [19, 20]. It showed a Cronbach’s alpha of 0.46 in this study.

The level of social support was assessed using the Oslo Social Support Scale (OSSS-3), a self-reported 3-item measure of the number of confidants, sense of concern from people, and relationship with neighbors for practical help. The scores range from 3–14 with 3–8 (poor support), 9–11 (moderate support), and 12–14 (strong support) [21]. It showed a Cronbach’s alpha of 0.53 in this study.

### Data handling and analysis

The data were entered, coded, checked, and cleaned using Epi-data version 3.1 and exported to SPSS version 26 for analysis. Descriptive analysis was used to assess sociodemographic and other study variables. Factors with P-value < 0.20 on bivariable logistic analysis were entered into multivariable logistic regression. Statistical significance was determined at p-value < 0.05. The strength of association was interpreted using odds ratio and 95% confidence interval.

### Ethical consideration

Ethical clearance was obtained from the Institutional Review Board (IRB) of Bahir Dar University (IRB code number: አህጤኢ ም/ቴ/ሽ/ዳ/1/799). Permission and support letters were secured from the Amhara National Regional State Health Bureau and the administration offices of the study Kebeles and Health Centers. Written informed consent was obtained from each respondent. The respondents were given the right to refuse to take part in the study as well as to withdraw at any time during the study. Refusal to participate did not result in loss of medical care provided or any other benefits. No name or identifying information was indicated on the questionnaire, and all subjects were assured of confidentiality. Study participants found to have severe psychological distress were referred to the psychiatry clinic.

## Result

### Socio-demographic data of participants

Data were collected and analyzed from 707 individuals living with chronic medical illness and 776 participants from the general community giving a response rate of 95.7%. The mean age in years in chronic illness and community participants was 43.77 (±17.1) and 35.14 (±14.9), respectively. The two groups were compared for differences in their basic socio-demographic characteristics (Table 1) and psycho-social factors (Table 2).

**Table 1:** Socio-demographic characteristics of chronic medical illnesses and general community participants in Mecha demographic research center, Ethiopia.

Variables	Community participants (n = 776)	Chronic illness participants (n = 707)	P- value
	n (%)	n (%)	
Sex			0.257
Female	430 (55.4)	371 (52.5)	
Male	346 (44.6)	336 (47.5)	
Age			<0.001
18–24	197 (25.4)	83 (11.7)	
25–34	247 (31.8)	169 (23.9)	
35–44	163 (21)	128 (18.1)	
≥45	169 (21.8)	327 (46.3)	
Marital status			0.002
Married	491 (63.3)	429 (60.7)	
Single	178 (22.9)	132 (18.7)	
Divorced	54 (7.0)	63 (8.9)	
Widowed	53 (6.8)	83 (11.7)	
Educational status			<0.001
Cannot read and write	196 (25.3)	373 (52.8)	
Primary school (1–8)	179 (23.1)	169 (23.9)	
Secondary school (9–12)	192 (24.7)	105 (14.9)	
Diploma and above	209 (26.9)	60 (8.5)	
Occupation			<0.001
Government employee	157 (20.2)	59 (8.3)	
Private employee	175 (22.6)	72 (10.2)	
Merchant	135 (17.4)	67 (9.5)	
Housewife	165 (21.3)	190 (26.9)	
Farmer	96 (12.4)	294 (41.6)	
Not having job	48 (6.2)	25 (3.5)	
Monthly income*			<0.001
< 600	216 (27.8)	171 (24.2)	
600–999	128 (16.5)	80 (11.3)	
1000–2500	203 (26.2)	349 (49.4)	
>2500	229 (29.5)	107 (15.1)	

* Monthly income (Ethiopian birr)

**Table 2:** Psycho-social factors among people living with chronic medical illness and general community participants in Mecha demographic research center, Ethiopia.

Variable	Community participants (n = 776)	Chronic illness participants (n = 707)	P-value
	n (%)	n (%)	
Family size			0.007
<5	580 (74.7)	484 (68.5)	
≥ 5	196 (25.3)	223 (31.5)	
Living condition			0.011
With family	636 (82.0)	619 (87.6)	
Alone	100 (12.9)	65 (9.2)	
Homeless/relatives	40 (5.1)	23 (3.3)	
Social support			<0.001
Poor	157 (20.2)	48 (6.8)	
Moderate	390 (50.3)	268 (37.9)	
Strong	229 (29.5)	391 (55.3)	
Stressful life events			<0.001
Yes	386 (49.7)	290 (41.0)	
No	390 (50.3)	417 (59.0)	
Family history of medical illness			0.047
Yes	181 (23.3)	135 (19.1)	
No	595 (76.7)	572 (80.9)	
Family history of mental illness			0.144
Yes	22 (2.8)	12 (1.7)	
No	754 (97.2)	695 (98.3)	

### Psychological distress among people living with chronic medical illness

The magnitude of psychological distress among people living with chronic medical illness was 62.0% (95% CI: 58.1, 65.6). It was found to be higher among people living with HIV/AIDS (75.2%) and those having two or more chronic medical diseases (71.4%) and hypertension (65.7%). For individuals with other types of chronic medical illnesses, the psychological distress estimates were epilepsy (59.8%), diabetes mellitus (56.0%), asthma (51.5%), and tuberculosis (32.6%). Being single (AOR: 1.70; 1.09, 2.64), divorced (AOR: 5.14; 2.40, 11.01), positive family history of chronic medical illness (AOR: 1.63; 1.06, 2.50), low social support (AOR: 1.51; 1.07, 2.12) and taking more than two types of drugs (AOR: 1.72; 1.23, 2.40) were statistically significantly associated with psychological distress among people living with chronic medical illness (Table 3).

**Table 3:** Bivariable and multivariable analysis of psychological distress among people living with chronic medical illness in Mecha demographic research center, Ethiopia (n = 707).

Variables	Category	Psychological distress		COR (95% CI)	AOR (95% CI)	P-value
		Yes	No			
**Marital status**	Married	242	187	1	1	
	Single	85	49	1.39 (0.93,2.09)	1.70 (1.09,2.64) *	0.018
	Divorced	54	9	4.63 (2.23,9.63)	5.14 (2.40,11.01) *	0.011
	Widowed	57	26	1.69 (1.02,2.79)	1.65 (0.96,2.82)	0.065
**Income (Ethiopian Birr)**	<600	95	76	0.90 (0.55,1.47)	0.67 (0.39,1.15)	0.152
	600–999	51	29	1.28 (0.70,2.32)	0.99 (0.52,1.89)	0.981
	1000–2500	230	119	1.40 (0.90,2.18)	1.23 (0.77,1.95)	0.387
	>2500	62	45	1	1	
**Occupation**	Government	35	24	1	1	
	Private	49	23	1.46 (0.71,2.99)	1.60 (0.72,3.56)	0.248
	Merchant	33	34	0.66 (0.32,1.35)	0.63 (0.30,1.32)	0.221
	Housewife	120	70	1.17 (0.64,2.14)	1.50 (0.72,3.12)	0.272
	Farmer	186	108	1.18 (0.66,2.09)	1.51 (0.75,3.02)	0.244
	Not on job	15	10	1.03 (0.39,2.67)	1.65 (0.56,4.86)	0.358
**Family medical illness**	Yes	96	39	1.65 (1.10,2.49)	1.63 (1.06,2.50) *	0.013
	No	342	230	1	1	
**Living condition**	Family	378	241	1	1	
	Alone	47	18	1.66 (0.94,2.93)	1.53 (0.75,3.08)	0.228
	Homeless/relatives	13	10	0.83 (0.36,1.92)	0.77 (0.30,1.99)	0.597
**Social support**	Poor/moderate	219	97	1.77 (1.29,2.42)	1.51 (1.07,2.12) *	0.018
	Strong	219	172	1	1	
**Types of drugs**	One	228	170	1	1	
	≥ two	209	99	1.57 (1.15,2.15)	1.72 (1.23,2.40) *	0.002
**Medication Duration**	0–5 years	374	219	1	1	
	> 5 years	62	50	0.72 (0.48,1.09)	0.70 (0.45,1.09)	0.144

* = p-value < 0.05(significant), 1 = Reference, COR = Crude odd ratio, AOR = Adjusted odd ratio

### Psychological distress in the general population

The magnitude of psychological distress in the general population was 35.1% (95% CI: 31.7, 38.4%). Being female (AOR:1.77;1.20,2.62), divorced (AOR:1.96;1.02,3.76), widowed (AOR:2.88;1.41,5.88), merchant occupation (AOR:2.20;1.28,3.81), family history of medical illness (AOR:1.58;1.08,2.31), family history of mental illness (AOR:5.82;1.97,17.22), stressful life events (AOR:2.40;1.72,3.36) and poor social support (AOR:1.65;1.01,2.68) were statistically significantly associated with psychological distress in the general population (Table 4).

**Table 4:** Bivariable and multivariable analysis of psychological distress in the general population in Mecha demographic research center, Ethiopia (n = 776).

Variables	Category	Psychological distress		COR (95%, CI)	AOR (95%, CI)	P-value
		Yes	No			
**Sex**	Male	102	244	1	1	
	Female	170	260	1.56 (1.16,2.11)	1.64 (1.20,2.56) *	0.008
**Age(years)**	18–24	61	136	0.40 (0.26,0.62)	0.62 (0.37,1.04)	0.139
	25–34	70	177	0.35 (0.24,0.53)	0.55 (0.33,0.91) *	0.004
	35–44	52	111	0.42 (0.27,0.66)	1.02 (0.54,1.89)	0.044
	≥45	89	80	1	1	
**Marital status**	Married	153	338	1	1	
	Single	53	125	0.94 (0.64,1.36)	1.17 (0.82,1.97)	0.464
	Divorced	30	24	2.76 (1.56,4.88)	1.96 (1.03,3.77) *	0.028
	Widowed	36	17	4.68 (2.55,8.58)	3.03 (1.53,5.98) *	0.008
**Income (Ethiopian Birr)**	<600	98	118	2.39 (1.60,3.56)	1.38 (0.78,2.46)	0.188
	600–999	36	92	1.12 (0.69,1.83)	0.88 (0.46,1.67)	0.861
	1000–2500	79	124	1.83 (1.23,2.76)	1.54 (0.94,2.52)	0.063
	>2500	59	170	1	1	
**Occupation**	Government	34	123	1	1	
	Private	67	108	2.44 (1.38,3.65)	1.50 (0.87,2.60)	0.288
	Merchant	57	78	2.64 (1.58,4.40)	2.20 (1.28,3.81) *	0.015
	Housewife	67	98	2.47 (1.51,4.04)	1.44 (0.81,2.55)	0.395
	Farmer	24	72	1.20 (0.66,2.19)	0.69 (0.34,1.41)	0.314
	Not on job	23	25	3.32 (1.68,6.58)	1.39 (0.62,3.12)	0.547
**Family history of medical illness**	Yes	88	93	2.11 (1.50,2.96)	1.58 (1.08,2.31) *	0.024
	No	184	411	1	1	
**Family history of mental illness**	Yes	17	5	6.65 (2.42,18.24)	5.82 (1.97,17.22) *	0.002
	No	255	499	1	1	
**Stressful life events**	Yes	98	292	2.44 (1.80,3.31)	2.40 (1.72,3.36) *	<0.001
	No	174	212	1	1	
**Social Support**	Poor	77	80	2.59 (1.69,3.97)	1.65 (1.01,2.68) *	0.045
	Moderate	133	257	1.39 (0.97,1.99)	1.11 (0.75,1.65)	0.599
	Strong	62	167	1	1	

* = p-value < 0.05(significant), 1 = Reference, COR = Crude odd ratio, AOR = Adjusted odd ratio

### Psychological distress comparison among people living with chronic medical illness and the general population

The burden of psychological distress was much higher in chronic medical illness study participants (62.0%, 95% CI, 58.7–65.5%) than the general population participants (35.1%, 95% CI, 31.7–38.7%). This difference was statistically significant (χ^2^ = 107.276, p < 0.001).

Males with chronic medical illness had psychological distress two times higher than those from the community (60.7% versus 29.5%). Besides, females in the chronic illness group had higher psychological distress than those in the community (63.1% versus 39.5%) (Table 5).

**Table 5:** Psychological distress distribution among people living with chronic medical illness and general community participants in Mecha demographic research center, Ethiopia.

Variable	Category	Psychological distress among community (n = 776)		Psychological distress among chronic illness (n = 707)	
		Yes, n (%)	No, n (%)	Yes, n (%)	No, n (%)
Family size	<5	206 35.5)	374 (64.5)	301 (62.2)	183 (37.8)
	≥ 5	66 (33.7)	130 (66.3)	137 (61.4)	86 (38.6)
Living condition	With family	228 (35.8)	408 (64.2)	378 (61.1)	241 (38.9)
	Alone	30 (30.0)	70 (70.0)	47 (72.3)	18 (27.7)
	Homeless/relatives	14 (35.0)	26 (65.0)	13 (56.5)	10 (43.5)
Social support	Poor	77 (49.0)	80 (51.0)	44 (91.7)	4 (8.3)
	Moderate	133 (34.1)	257 (65.9)	175 (65.3)	93 (34.7)
	Strong	62 (27.1)	167 (72.9)	219 (56.0)	172 (44.0)
Stressful life events	Yes	174 (45.1)	212 (54.9)	179 (61.7)	111 (38.3)
	No	98 (25.1)	292 (74.9)	259 (62.1)	158 (37.9)
Family history of medical illness	Yes	88 (48.6)	93 (51.4)	96 (71.1)	39 (28.9)
	No	184 (30.9)	411 (69.1)	342 (59.8)	230 (40.2)
Family history of mental illness	Yes	17 (77.3)	5 (22.7)	9 (75.0)	3 (25.0)
	No	255 (33.8)	499 (66.2)	429 (61.7)	266 (38.3)

## Discussion

This study demonstrated that the burden of psychological distress was much higher among the chronic medical illness participants (62.0%, 95% CI, 58.7–65.5%) than the general population participants (35.1%, 95% CI, and 31.7–38.7%). It also indicated that this difference was statistically significant between the two groups. The odds of psychological distress among people with chronic medical illness was 3.2 times higher than the community sample. This was in line with and supported by a study that showed a three times higher risk of psychological distress among individuals with comorbid chronic medical illness than participants with no comorbid chronic medical illness [22]. The relationship between chronic disease and mental illness is generally thought to be bidirectional. People living with a serious mental illness are at higher risk of experiencing a wide range of chronic physical conditions. Conversely, people living with chronic medical conditions experience psychological distress twice the rate of the general population [23].

Among people living with chronic medical diseases, the study showed findings similar with those of other studies carried out in Ethiopia (63.3%) [24], Pakistan (60.2%, [25], Nigeria (64.4%) [26], and Delhi India (58.7%) [27]. However, the magnitude of psychological distress in this study was higher than that in studies done in Southern Ethiopia (39.2%) [28], Addis Ababa, Ethiopia (53.1%) [29], India (50.8%) [30], Kenya (42.3%) [31] and Iran (14%) [32]. These variations might possibly be a result of differences in sample size, inclusion criteria, screening instrument used to assess psychological distress, and the difference in the study population. In India, only 130 participants living with diabetes or hypertension were included in the study and they used General Health Questionnaire (GHQ 12) to assess psychological distress. The two studies from Ethiopia used Self-Reporting Questionnaires (SRQ-20).

The magnitude of psychological distress among the general population in this study was higher than in other community studies done in different parts of Ethiopia: Harari Regional State (14.9%), Addis Ababa (17.7%), Jimma Town (22.7%), and Illu Ababore Zone (27.2%) [33–36]. This might be due to the differences in screening tool (Self-Reporting Questionnaire-20), study period, and sociodemographic characteristics. For instance, the proportion of female participants was higher in this study than in the study in Harari Regional State (55.4% versus 38.1%). In that study, women had a higher magnitude of of depression and anxiety than men [37].

On the other hand, the psychological distress in the current study was lower than that in a study in Silte Zone residents of Ethiopia (39.7%) [38]. This might be due to the fact that none of the study participants in Silte Zone had strong social support. In the current study, 29.5% had strong social support. Low social support is known to be associated with the risk of developing psychological distress or worsening of an already existing mental health problem [39]. An other possible explanation for the difference is that the proportion of respondents having a family history of mental illness in the current study was lower (2.8% versus 49.8%).

From the multivariable logistic regression analysis among chronic medical illness study participants, it was found that individuals who were single or divorced, having family history of medical illness, low social support, and taking two or more types of drugs were likely to have psychological distress. The result was consistent with findings of other studies [22, 28, 40]. Marriage is the central part of all human society and its effects on mental illness are often identified as a key component [41]. Research consistently has shown that divorced/ widowed marital status was positively associated with smoking, poor quality of life, high psychological distress, anxiety, and depression [42]. Being married brings social support which is a very crucial role in an individual’s life satisfaction and lack of social support can be a predictor of psychological problems relating to low self-esteem, loneliness, anxiety, and depression [43]. A systematic review and meta-analysis among people living with HIV/AIDS in Ethiopia showed a higher odds of depression among individuals with poor social support than those who had strong social support [44].

On the other hand, the study indicated that being female, divorced or widowed marital status, family history of medical illness, family history of mental illness, stressful life events, and low social support were factors significantly associated with psychological distress among community participants. This result was also consistent with findings of previous studies [35, 36, 38, 40, 45]. Disrupted marital status, low social support, having a family history of mental illness and stressful life events were strong risk factors to develop psychological distress. Family history of mental illness and stressful events in daily life significantly predict the onset and relapse of psychological distress [46–48]. The rise of psychological distress in late-life mostly reflects losses in marriage, employment, functional limitations, chronic disease burden, denied their wisdom and power [49–51].

### Implication of the study

The study found a high magnitude of psychological distress among people with chronic medical conditions. This undiagnosed and untreated high psychological distress may lead to non-adherence to treatment and poor control of medical conditions. Studies showed that untreated psychological distress resulted in poor control of sugar level in diabetes mellitus [12]; worse treatment outcomes, poorer quality of life and greater disability in tuberculosis [13]; uncontrolled seizure in epilepsy [14]; and high blood pressure in hypertension [15]. The findings of this study increase the awareness among health care providers of the high magnitude of psychological distress among people living with chronic medical conditions so that its diagnosis and treatment may lead to improved treatment outcomes and quality of life of such cases.

### Strengths and limitations of the study

To the best knowledge of the authors, this is the first study that compared psychological distress between people living with chronic medical diseases and the general population in Northwest Ethiopia. The other major strength of the study was that the outcome variable was assessed by using locally validated instrument having very good psychometric properties, Kessler-10. An important limitation of the study was that screening for chronic medical conditions like HIV, diabetes mellitus and hypertension was not done in the general community. In addition, authors want to stress that the tools used to measure stressful life events and social support have been found to have low Cronbach’s alpha. This might partly be due to the fact that the tools have few items. Scales with short list of items have difficulties in achieving high Cronbach’s alpha for it strongly depends on the number of items. Shorter scales may be considered reliable even when Cronbach’s alpha is below 0.7 which may be as low as 0.5 for a three-item scale [52]. Generally, we recommend readers of this paper to remain cognizant of the problematic points behind the interpretation and effects of low values of Cronbach’s alpha as a measure of reliability of scales.

## Conclusion

Compared to the general population, people living with chronic medical illnesses had a high magnitude of psychological distress. Marital status, family history of medical illness, and low social support were statistically significant predictors of psychological distress in both comparative groups. The emotional dimension of chronic medical illnesses should always be given due consideration during the provision of medical care. Future studies should focus on the impact of diagnosis and treatment of psychological distress on the outcome and quality of life of people living with chronic medical illness.

## Supporting information

S1 Table Bivariable and multivariable logistic analysis of variables associated with psychological distress among all study participants in Mecha demographic research center, Ethiopia. (DOCX)
