# Supplementary material for: Psychological distress among people living with chronic medical illness and the general population, Northwest Ethiopia: A comparative cross-sectional study
Source: PLoS One. 2022 Dec 1;17(12):e0278235. doi: 10.1371/journal.pone.0278235 (PMC9714906; doi:10.1371/journal.pone.0278235)
Supplement: S1 Table — (DOCX) [file pone.0278235.s001.docx]

S1 Table. Bivariable and multivariable logistic analysis of variables associated with psychological distress among all study participants in Mecha demographic research center, Ethiopia

| Variable | Category | Psychological  Distress | | COR (95% CI) | AOR (95% CI) | P-value |
| --- | --- | --- | --- | --- | --- | --- |
|  |  | Yes | No |  |  |  |
| Sample type | Health facility | 438 | 269 | 3.02 (2.44,3.73) | 3.22 (2.49,4.16) * | <0.01 |
|  | Community | 272 | 504 | 1 | 1 |  |
| Sex | Male | 306 | 376 | 1 | 1 |  |
|  | Female | 404 | 397 | 1.25 (1.02,1.53) | 1.27 (1.01,1.61) * | 0.04 |
| Residence | Urban | 472 | 629 | 0.45 (0.35,0.57) | 1.12 (0.80,1.57) |  |
|  | Rural | 238 | 144 | 1 | 1 |  |
| Age(years) | 18-24 | 110 | 170 | 0.44 (0.33,0.60) | 0.93 (0.59,1.45) |  |
|  | 25-34 | 172 | 244 | 0.48 (0.37,0.63) | 0.80 (0.57,1.12) |  |
|  | 35-44 | 134 | 157 | 0.58 (0.44,0.78) | 0.94 (0.66,1.32) |  |
|  | >45 | 294 | 202 | 1 | 1 |  |
| Educational status | Cannot read and write | 343 | 226 | 3.34 (2.45,4.54) | 1.90 (1.27,2.85) * | <0.01 |
|  | From grade 1-8 | 172 | 176 | 2.15 (1.54,3.00) | 1.59 (1.08,2.35) * | 0.02 |
|  | From grade 9-12 | 111 | 186 | 1.31 (0.93,1.86) | 1.02 (0.69,1.52) |  |
|  | Diploma and above | 84 | 185 | 1 | 1 |  |
| Marital status | Married | 395 | 525 | 1 | 1 |  |
|  | Single | 138 | 172 | 1.06 (0.82,1.38) | 1.35 (0.99,1.84) |  |
|  | Divorced | 84 | 33 | 3.38 (2.21,5.16) | 2.65 (1.66,4.23) * | <0.01 |
|  | Widowed | 93 | 43 | 2.87 (1.96,4.22) | 1.88 (1.23,2.87) * | <0.01 |
| Income  (Ethiopian Birr) | <600 | 193 | 194 | 1.77 (1.31,2.38) | 0.75 (0.52,1.10) |  |
|  | 600-999 | 87 | 121 | 1.28 (0.89,1.82) | 0.58 (0.38,0.91) * | 0.02 |
|  | 1000-2500 | 309 | 243 | 2.26 (1.71,2.98) | 1.11 (0.79,1.54) |  |
|  | >2500 | 121 | 215 | 1 | 1 |  |
| Occupation | Government | 69 | 147 | 1 | 1 |  |
|  | Private | 116 | 131 | 1.88 (1.29,2.76) | 1.15 (0.64,2.08) |  |
|  | Merchant | 90 | 112 | 1.71 (1.15,2.55) | 1.36 (0.76,2.42) |  |
|  | Housewife | 187 | 168 | 2.37 (1.66,3.37) | 1.05 (0.56,1.98) |  |
|  | Farmer | 210 | 180 | 2.48 (1.75,3.52) | 1.02 (0.54,1.90) |  |
|  | Not on job | 38 | 35 | 2.31 (1.35,3.97) | 1.20 (0.56,2.55) |  |
| Family history of medical illness | Yes | 184 | 132 | 1.69 (1.32,2.18) | 1.78 (1.35,2.35) * | <0.01 |
|  | No | 526 | 641 | 1 | 1 |  |
| Family history of mental illness | Yes | 26 | 8 | 3.63 (1.63,8.08) | 3.69 (1.56,8.70) * | <0.01 |
|  | No | 684 | 765 | 1 | 1 |  |
| Stressful life events | Yes | 357 | 450 | 0.73 (0.59,0.89) | 1.37 (1.09,1.73) * | <0.01 |
|  | No | 353 | 323 | 1 | 1 |  |
| Social  Support | Poor | 121 | 84 | 1.74 (1.26,2.39) | 2.47 (1.68,3.63) * | <0.01 |
|  | Moderate | 308 | 350 | 1.06 (0.85,1.32) | 1.30 (1.01,1.68) |  |
|  | Strong | 281 | 339 | 1 | 1 |  |

* = p-value < 0.05(significant), 1= Reference, COR= Crude odd ratio, AOR= Adjusted odd ratio
